# Ultrasound for predicting difficult airway in obstetric anesthesia: Protocol and methods for a prospective observational clinical study

**DOI:** 10.1097/MD.0000000000017846

**Published:** 2019-11-15

**Authors:** Bi-Xin Zheng, Huan Zheng, Xue-Mei Lin

**Affiliations:** a Key Laboratory of Birth Defects and Related Diseases of Women and Children (Sichuan University), Ministry of Education; b Department of anesthesiology, West China second university hospital; c Department of Pain Management, West China hospital, Sichuan University; d Department of anesthesiology, Sichuan provincial people's hospital, Chengdu, China.

**Keywords:** difficult airway, obstetric anesthesia, ultrasound

## Abstract

**Background:**

Failed intubation and ventilation during cesarean deliveries are important causes of anesthetic-related maternal mortality. Due to the physiological changes in airway anatomy, parturient had higher incidences of difficult airway than non-obstetric population. Accurate airway assessment is the first step and the most important in airway management. However, the common clinical screening tests, shown low sensitivity and specificity with a limited predictive value. Ultrasound is a quick, noninvasive, inexpensive tool, with the advancement of ultrasound technology, modern ultrasound machine is more portable with better resolution and enhanced tissue penetration, provide better imaging in tissues like epiglottis, vocal cords, ring-shaped membrane, and can be used in airway assessment. Here, the aim of the current study was to find whether preoperative ultrasound assessment of neck anatomy can predict difficult airway in parturient, and provide new ideas and a theoretical basis in the airway management of obstetric anesthesia.

**Methods:**

This is a prospective, observational single-blinded study in a single-center. Subjects will be recruited from patients aged from 18 to 60 years, gestational age ≥ 36 weeks, scheduled for cesarean section under general anesthesia and tracheal intubation. Ultrasound measurement will be performed to detect anterior cervical soft tissue thickness at five anatomical levels (hyoid bone, epiglottis, cricothyroid membrane, thyroid isthmus and suprasternal notch) in the upper airway. The thickness of the soft tissue in the front of the neck and clinical airway measurements will be compared between the “easy intubation” and “difficult intubation” group divided by Cormack–Lehane grade. Receiver-operating characteristic curves were used to determine the sensitivity and specificity of “difficulty prediction capability” of each sonographic and physical measurements. Clinical factors associated with difficult intubation will be determined by univariate analyses. Multiple logistic regression analysis performed to determine independent predictors of difficult intubation.

**Conclusions:**

The study outlined in this protocol will explore the possibility of ultrasound for predicting difficult airway in obstetric anesthesia. This may provide new insight into the practice of airway management.

**Trial registration:**

Chinese Clinical Trial Registry, ChiCTR1800018949.

## 1 Introduction

In obstetric anesthesia, difficult airway is frequently unexpected. Failed intubation and ventilation during cesarean deliveries are important causes of anesthetic-related maternal mortality.^[1,2]^ Due to the physiological changes in airway anatomy like tracheal edema and obesity, parturient had higher incidences of difficult airway than non-obstetric population. The statistics in the USA indicated the most deaths of parturient were attributed to airway management difficulties, failure to provide adequate oxygenation and carbon dioxide removal, lead to life-threatening complications.^[3]^ With the implementation of the “2-child policy” in China, the number of elderly and critically ill women has increased dramatically,^[4]^ more parturient need to receive cesarean section with general anesthesia, that increases the risk of a difficult airway and maternal mortality.

Airway assessment is an essential component for the anesthesiologist. Accurate airway assessment is the first step towards avoiding potential complications and should always be the most important in airway management. Available guidelines suggest the clinical evaluation of Mallampati classification, thyromental distance, upper lip bite test, interincisor distance, BMI could be used to predict the risk of difficult airway.^[5,6]^ However, despite these clinical tests, showed low sensitivity and specificity with a limited predictive value, especially if only a single assessment method is used.^[7,8]^

During the last few years, perioperative ultrasound has been widely used in anesthesiology and intensive care practice for diagnostic and therapeutic relevance, such as nerve block, vascular cannulation, and focused cardiovascular ultrasound examination (FoCUS).^[9,10]^ With the advancement of ultrasound technology, modern ultrasound machine is more portable with better resolution and enhanced tissue penetration, provide better imaging in tissues like epiglottis, vocal cords, ring-shaped membrane. Ultrasound is a quick, noninvasive, inexpensive tool and can provide accurate information in airway assessment. With characteristics of non-radiating is the best application in obstetric anesthesia compared with computerized tomography (CT) and X-ray. It has been reported in the literature that ultrasound can help determine the position of the tracheal tube and evaluate the position of the laryngeal mask and predict difficult airways.^[11]^ Recently, studies had reported that ultrasound can be taken into consideration as a predictor of difficult laryngoscopy by measuring the anterior neck soft-tissue thickness.^[12,13]^

However, there is limited data to provide high-level scientific evidence of the potential role of ultrasound on assessing the airway in parturient. The methodologically rigorous trials are important to determine the use of ultrasound in predicting difficult airway, and to provide new ideas and a theoretical basis in the airway management of obstetric anesthesia.

## 2 Objective

The aim of the current study was to find whether preoperative ultrasound assessment of neck anatomy can predict difficult airway in parturient, by analysis correlations between ultrasound measurements of anterior cervical soft tissue thickness at 5 anatomical levels (hyoid bone, epiglottis, cricothyroid membrane, thyroid isthmus and suprasternal notch) in the upper airway, and the Cormack–Lehane grade. The secondary objective was to determine whether clinical screening tests are independent predictors of difficult airway.

We consider that ultrasound airway assessment have higher specificity and sensitivity than traditional clinical test in predict difficult airway.

## 3 Methods

### 3.1 Ethics approvals and registration

Ethic approval for this study has been obtained from the Medical Ethics Committee of Sichuan Academy of Medical Sciences & Sichuan Provincial People's Hospital (No. 2018-240). The trial was registered at Chinese Clinical Trial Registry (ChiCTR1800018949). Participating sites obtained written informed consent by participating patients or their legal surrogates in accordance with local/ national legislations. Fully data will be upload on the Clinical Trial Management Public Platform. Results will be published in peer-reviewed journals and disseminated at international conferences.

### 3.2 Study design

**Figure 1:**
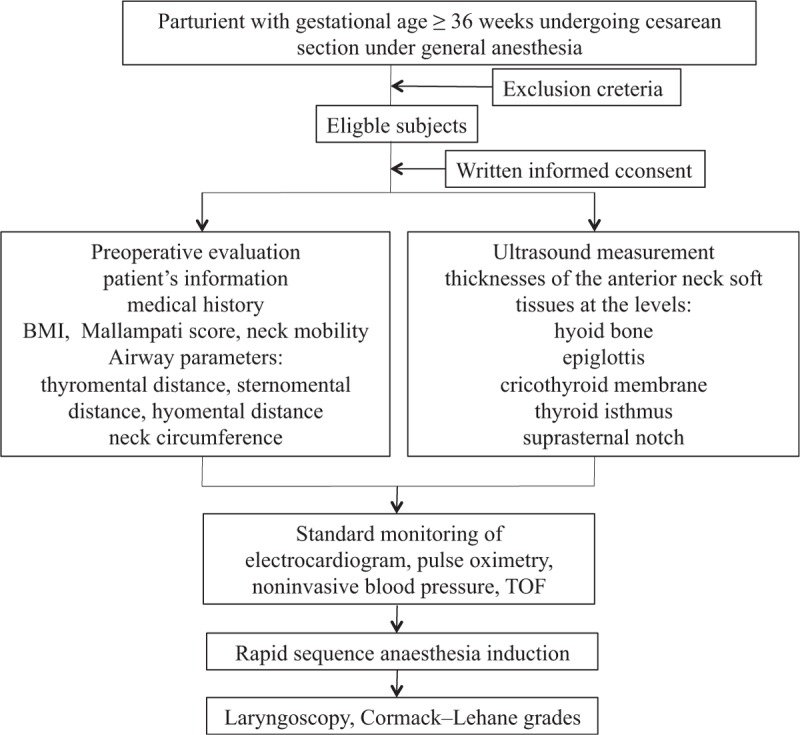
Flow chart of the study.

The flowchart of this trial is in Figure 1. This is a prospective, observational single-blinded study being conducted in a single center. The study will observe patients scheduled for cesarean section under general anesthesia in the operating theatre of the Sichuan Provincial People's Hospital, Chengdu, China from January 2020 to January 2021.

### 3.3 Recruitment of patients and blinding

The subjects will be recruited from the department of obstetrics surgical ward in Sichuan Provincial People's Hospital. Patients were included in the study after obtaining informed consent. Both Chinese and English written informed consent in will be obtained from every participant. Once participants were enrolled in the study, they will be anonymized by an identification code. The subjects can voluntarily withdraw from the trial at any time. Preoperative evaluation and ultrasound examination will be performed by a single trained investigator skilled with airway ultrasound examination. The anesthesiologists perform the laryngoscopy and endotracheal intubation, they will be blinded to the findings of ultrasound examination.

**Inclusion criteria are:**

- Patients aged from 18 to 60 years.
- Live fetus at gestational age ≥ 36 weeks.
- Singleton or multiple pregnancy.
- Mentally competent.
- American Society of Anesthesiologists (ASA) physical status I–III.
- Subject is planning cesarean section under general anesthesia and tracheal intubation at our maternity at Sichuan Provincial People's Hospital.
- Informed and written consent.

**Exclusion criteria are:**

- Abnormal pharynx or anatomy.
- Expected difficult laryngoscopy with cervical spine abnormality, maxillofacial anomalies, upper airway disease, a history of previous difficult intubation.
- Pregnancies ending with a stillborn.
- Women presenting for the study but who have been previously included in the study in a previous pregnancy.
- Women who are unconscious, severely ill, those with learning difficulties, or mentally handicapped.

### 3.4 Outcome measures

Primary outcomes:

- Ultrasound measurements: thickness of anterior cervical soft tissue at five levels: distance (cm) from skin to the trachea at hyoid bone, epiglottis, cricothyroid membrane, thyroid isthmus, suprasternal notch level.
- Cormack–Lehane grade

Secondary outcomes:

Body mass index (BMI), Mallampati score, neck mobility (flexion/extension degrees from midline), upper lip bite test, jaw movement, mouth opening (cm), thyromental distance (cm), sternomental distance (cm), hyomental distance (cm) and neck circumference (cm) at the level of the thyroid cartilage.

### 3.5 Preoperative evaluation and ultrasonography

Preoperative evaluation and ultrasound examination were performed one day before the surgery, data was obtained in standard fashion. The patient's information, medical history was obtained. Physical examination include height, weight, body mass index (BMI). Airway evaluation include Mallampati score, neck mobility (flexion/extension degrees from midline), upper lip bite test, jaw movement, prominent incisor. A flexible ruler will be used to measure airway parameters, mouth opening (cm), thyromental distance (cm), sternomental distance (cm), hyomental distance (cm) and neck circumference (cm) at the level of the thyroid cartilage.

**Figure 2:**
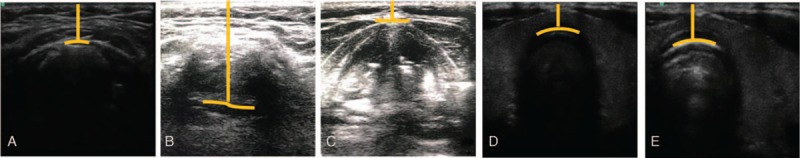
Ultrasound measurement the anterior cervical soft tissue thickness at five anatomical levels (A) hyoid bone, (B) epiglottis, (C) cricothyroid membrane, (D) thyroid isthmus, (E) suprasternal notch. Consent to publish the figure was obtained from the patient.

The thicknesses of the anterior neck soft tissues were measured with a portable ultrasound machine (M7 Super; Mindray, Shenzhen, China). A 6 to 13 MHz high frequency curvilinear ultrasound transducer was used for ultrasound scanning. Patients will be explained about the ultrasonography procedure and positioned in supine with the head in a neutral position. Ultrasonography with the probe placed in the transverse axis, craniocaudal sagittal scan, submental region of the neck. The distances were measured at five levels (hyoid bone, epiglottis, cricothyroid membrane, thyroid isthmus and suprasternal notch) with normal and extended neck position in the median axis. Figure 2A–E showed the ultrasonography procedure:

A. measure the distance from the skin to the hyoid bone;
B. distance from the skin to the epiglottis performed at the thyrohyoid membrane level;
C. distance from skin to anterior commissure of the cricothyroid membrane;
D. distance from skin to thyroid isthmus;
E. distance from skin to the trachea at suprasternal notch level. The images will be retrieved from ultrasound machine and stored on computer.

### 3.6 Anesthesia procedure

On the operation day, standard monitoring of electrocardiogram, pulse oximetry, noninvasive blood pressure will be performed throughout anesthesia to detect clinical vital signs. Train-of-four (TOF) were used for neuromuscular monitoring. Patients will fasting for 8 hours, clear fluids may be given up to 2 hours preoperatively before anesthesia. Ranitidine (150 mg), a H2-receptor antagonist was administrated the night before and two hours before anesthesia for stomach preparation. Equipment for managing the difficult airway will be ready for use. General anesthesia was administered after the disinfection and towel laying procedures, obstetricians are standby for cesarean section. Rapid sequence anesthesia induction was recommended for obstetric anesthesia and will be performed as standard protocol.^[14]^ Preoxygenation was performed for 3 min through a face mask, propofol (2 mg/kg) was administered, then a neuromuscular blocker (rocuronium 1 mg/kg) was given.

### 3.7 Laryngoscopy

When the anesthetic depth was enough, and TOF indicated neuromuscular blockade was deemed as adequate (TOF 1/4 0), the anesthesiologists with more than 5 years of experience perform the laryngoscopy and endotracheal intubation. Patients will be in a standard sniffing position, Macintosh curved laryngoscope blade will be used to perform direct laryngoscopy and intubation. A modified version of the Cormack–Lehane grade will be used to evaluate difficult airway.^[15]^ The Cormack–Lehane grades 1 or 2a will be designated as “easy”, grades 2b or 3a, and grades 3b or 4 represent “restricted or difficult” laryngoscopies: grade 2b (only arytenoids visible), 3a (only epiglottis visible and liftable), 3b (epiglottis adherent to pharynx) and 4 (no laryngeal structures seen). A maximum of three attempts will be permitted. In addition, if the SpO2 decrease to less than 90% or patients with unanticipated difficult ventilation or intubation will be withdrawn from the study. Steps followed the process in the guidelines of the Obstetric Anesthetists’ Association and the Difficult Airway Society will be taken immediately.^[6,14]^ LMA will be utilized to maintain ventilation. If the SGA failed, cricothyroid membrane puncture will be performed immediately. The proper capnograph waveform could confirm the tracheal intubation. The attending anesthesiologist performs the further anesthetic management of the patient.

### 3.8 Data collection

Data collected in the study are listed in Table 1. Electronic data will be stored and saved on a pass-word-protected computer. Hard (paper) copies of the consent form, questionnaire, and data sheets from the ultrasound examination will be stored in a locked filing cabinet in the principal researcher's computer. Downloaded from and members of the research team will be given access to the data.

**Table 1:**
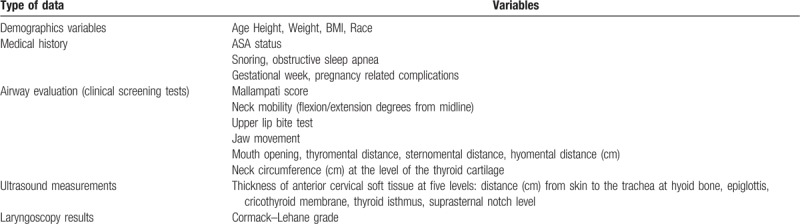
Data collected in the study.

### 3.9 Sample size and study power

Optimal sample size has been evaluated based on a statistical power of 80% and a significance level of 0.05 (two-tailed). According to the previous study, in obese surgical patients, an incidence of difficult intubation was about 10% to 20%.^[16,17]^ Ultrasound measurements should predict at least 80% difficulty intubations (Cormack–Lehane grade ≥ 2b), additionally, 20 events are needed to derive two or more correlates in a logistic regression model.^[18]^ It is estimated that a sample size of 106 will be needed, to adjust the possibility 20% of dropout, 127 subjects will be recruited into the study.

### 3.10 Statistics

After the intubation is complete, all data will be collected. Data analysis was performed using the statistical software SPSS 20.0. The thickness of the soft tissue in the front of the neck and clinical airway measurements will be compared between the “easy intubation” and “difficult intubation” group. ROC curves were used to determine the sensitivity and specificity of “difficult prediction capability” of each sonographic and physical measurements. Clinical factors associated with difficult intubation will be determined by univariate analyses. X^2^ analysis will be used for categorical or binary variables, unpaired t-test analysis was used for continuous variables. Multiple logistic regression analysis performed to determine independent predictors of difficult intubation. The results were averaged, and the data are presented as mean ± SD for each variable with continuous data. Categorical data were expressed as frequencies (%). All variables with P value along with their respective odds ratio and 95% confidence intervals were obtained. *P* value <.05 will be considered statistically significant.

## 4 Discussion

The maternal mortality has been reduced over the past four decade, but, the rate of failed tracheal intubation has remained unchanged in obstetrics anesthesia. Parturient have high risk of difficult airway due to the physically airway anatomy changes in the upper respiratory tract. Like obese patients, decreased functional residual capacity and increased oxygen requirements accelerate the onset of desaturation during apnea.^[1]^ In 2015, China's one-child policy was replaced by a universal two-child policy. Sichuan Provincial People's Hospital is the center of clinical service and research, one of National Top Hundred Excellent Hospitals in China. Since 2015, more elderly and critically ill parturient were received in our hospital, and need to cesarean section with general anesthesia. Accurate airway assessment is the first step towards avoiding potential complications and should always be the most important in airway management. Unfortunately, current clinical predictors such as the Mallampati score, thyromental distance had low sensitivity and high variability for detecting a difficult airway. Ultrasound is a high-frequency sound wave, it is bedside, radiation-free, cheap, fast and accurate, is the best choice in obstetric anesthesia. Previous study found that ultrasound distances from the thyroid isthmus to skin surface, the minimum distance from the hyoid bone to skin surface, the minimum distance from skin to anterior commissure of the vocal cords, the minimum distance from skin to trachea at the level of the jugular notch and the distance from skin to epiglottis midway can predict the difficult airway in non-obstetric patients.

This is likely the first study to find whether preoperative ultrasound assessment of neck anatomy can predict difficult airway in parturient. The limitations are,

1) in order to ensure patient safety, patients with unexpected and complicated difficult airway management will be withdrawn from the study, that might cause a potential selection bias.
2) this is a single-center observation study in Chinese people, the extrapolation of our findings to other centers and race of people needs further evaluation.

Our study will analysis correlations between ultrasound measurements of anterior cervical soft tissue thickness at five anatomical levels (hyoid bone, epiglottis, cricothyroid membrane, thyroid isthmus and suprasternal notch) in the upper airway, and the Cormack–Lehane grade, to provide a new basis for evaluation of difficult airways and to explore new and more effective methods to address this problem.

## Author contributions

ZB-X and ZH conceived the study and drafted the study design under the supervision of LX-M. All authors contributed to the conception, design and development of the study protocol. LX-M provided her expertise for the study design, data management and analysis. All authors approved the final version and agree to be accountable for the contents and integrity of this manuscript.

## Acknowledgments

We wish to acknowledge the patient cooperates with us to complete the ultrasound image acquisition, and consent for publication.
